# Knowledge, Attitudes, and Treatment Protocols for Helicobacter pylori Infection Among General Practitioners in Primary Healthcare Centers in Riyadh, Saudi Arabia

**DOI:** 10.7759/cureus.85492

**Published:** 2025-06-07

**Authors:** Waqar Farooqi, Raghad S Ahmed, Joud M Asfari, Shams M Alotaibi, Lean F Bander, Shaden S Almutiri, Abdulrahman A Almasood, Nawaf M Alsaggaf, Haitham S Alsugair

**Affiliations:** 1 Department of Internal Medicine, Almaarefa University, Riyadh, SAU; 2 College of Medicine, Almaarefa University, Riyadh, SAU

**Keywords:** antibiotic resistance, clinical guidelines, cross-sectional study, general practitioners, helicobacter pylori, knowledge, primary healthcare, public health, saudi arabia, treatment practices

## Abstract

Background

*Helicobacter pylori* is very common worldwide and is a major factor in causing peptic ulcers and stomach cancer. As frontline healthcare providers, general practitioners (GPs) play a vital role in the diagnosis and management of *H. pylori* infection. However, inconsistencies in knowledge and adherence to treatment guidelines remain a concern in many healthcare settings.

Objective

This study aimed to evaluate the knowledge, attitudes, and treatment practices of GPs regarding *H. pylori* infection in primary healthcare centers (PHCs) in Riyadh, Saudi Arabia.

Methods

A cross-sectional, questionnaire-based survey was conducted among 91 GPs working in both government and private PHCs. Data on demographics, knowledge, attitudes, and clinical practices were collected and analyzed using IBM SPSS Statistics for Windows, Version 26.0 (Released 2018; IBM Corp., Armonk, NY, USA). Chi-square tests were used to assess associations, with p-values <0.05 considered statistically significant.

Results

Among the respondents, 62.6% believed their knowledge of *H. pylori* was up to date, and 76.9% reported managing *H. pylori* cases. Triple therapy was the most commonly prescribed first-line treatment (68.1%), followed by quadruple therapy (29.7%). While 59.3% adhered to the recommended 14-day regimen, 35.2% still prescribed shorter courses. Significant associations were found between years of experience and knowledge level (p = 0.042), as well as between experience and choice of treatment regimen (p = 0.001). GPs in private clinics were more likely to treat *H. pylori* (p = 0.006) and more frequently used quadruple therapy (p = 0.007).

Conclusions

Although most GPs demonstrated awareness and a willingness to treat *H. pylori*, notable variations in knowledge and clinical practice persist. Clinical experience and healthcare settings significantly influence treatment decisions. These findings underscore the need for continuous professional development and the adoption of standardized treatment protocols in primary care.

## Introduction

*Helicobacter pylori *remains one of the most widespread bacterial infections globally, affecting approximately half of the world’s population [1]. A 2015 meta-analysis estimated that around 4.4 billion people are infected, with the highest prevalence in developing regions such as Africa, Latin America, and Asia [1]. In many low- and middle-income countries, *H. pylori *prevalence can exceed 80% among middle-aged adults [2]. The Middle East carries a particularly high burden; for instance, a seroepidemiological study in Jordan reported a prevalence of approximately 88% [3]. In Saudi Arabia, infection rates are also substantial, although they vary across studies and populations. Estimates suggest that 50-80% of adults with gastrointestinal symptoms may harbor *H. pylori *[2], and even healthy populations demonstrate significant levels of exposure.

These statistics underscore the public health importance of *H. pylori*, both globally and within the region. The bacterium is a well-established cause of chronic gastritis and peptic ulcer disease and has been classified as a Group 1 carcinogen due to its strong association with gastric malignancies [4,5]. Gastric cancer remains a major health concern worldwide, causing more than 750,000 deaths in 2020, making it the fourth leading cause of cancer-related mortality globally and the fifth in 2022 [6]. Such epidemiological trends reinforce the urgency of controlling *H. pylori *as a public health priority.

Although the exact modes of *H. pylori *transmission are not fully understood, oral-oral and fecal-oral routes are considered the most likely [4,7]. Factors such as overcrowding, poor sanitation, and unsafe water sources contribute significantly to the spread of infection, helping explain the higher prevalence in lower socioeconomic settings [3,4]. Studies in Africa and Asia have linked high endemicity to communal living conditions and contaminated food and water sources [3,7]. Conversely, improved hygiene and living conditions in higher-income countries have contributed to declining infection rates [7].

Despite its high prevalence, awareness and understanding of *H. pylori *remain inadequate in many communities. A large proportion of individuals are asymptomatic carriers and may remain undiagnosed. Cultural beliefs and barriers to healthcare access also shape attitudes toward the infection. For example, a survey in the United Arab Emirates found that 39% of people with *H. pylori*-related symptoms did not seek medical care, often opting for herbal remedies or citing concerns about treatment costs [8]. Similarly, a study in China found that although 41% of participants tested positive for *H. pylori*, 86% were unaware of their infection status [9]. These findings reveal critical gaps in public awareness, which may delay healthcare seeking and hinder efforts to reduce transmission. Enhancing public knowledge of *H. pylori *and its potential consequences is therefore essential.

From a clinical perspective, effective management of *H. pylori* involves timely diagnosis and adherence to evidence-based treatment protocols. Several diagnostic options are available. Noninvasive tests, such as the urea breath test and stool antigen test, are highly accurate and recommended for initial screening in primary care settings [4]. These tests are safe, convenient, and cost-effective for detecting active infections. Invasive methods - including endoscopic biopsy for rapid urease testing, histology, or culture - are generally reserved for patients undergoing endoscopy due to ulcer disease or alarm features (e.g., gastrointestinal bleeding or unexplained weight loss). Although culture is not routinely performed, it is valuable for determining antibiotic susceptibility in refractory cases [4].

International guidelines, including the Maastricht VI/Florence Consensus and various regional protocols, provide clear recommendations for eradication therapy [10,11]. Traditionally, the first-line treatment involves a proton pump inhibitor (PPI) combined with two antibiotics for 14 days - most commonly a clarithromycin-based triple regimen (PPI + clarithromycin + amoxicillin or metronidazole) - which can achieve cure rates exceeding 80% in susceptible strains [10,11]. However, rising antibiotic resistance over the past two decades has significantly reduced the efficacy of standard regimens. In 2017, the World Health Organization designated clarithromycin-resistant H. pylori as a high-priority pathogen due to its potential for treatment failure [11]. A global meta-analysis revealed that primary clarithromycin resistance now exceeds 15% in most regions, alongside high resistance rates to metronidazole and levofloxacin [11]. These resistance patterns undermine the success of empirical triple therapy.

As a result, the American College of Gastroenterology (ACG) and other bodies now recommend quadruple therapy as the preferred first-line approach in many regions [10]. This includes bismuth-based quadruple therapy (a PPI, bismuth, tetracycline, and metronidazole) or non-bismuth-based regimens using a PPI plus three antibiotics. Clarithromycin-based therapies are now typically reserved for populations with low resistance rates and are usually accompanied by a post-treatment test to confirm eradication. Adherence to current treatment guidelines and principles of antibiotic stewardship is essential to achieving high cure rates and preventing the further development of resistant *H. pylori *strains.

General practitioners (GPs) play a central role in the early identification and management of *H. pylori *infection. They are often the first point of contact for patients presenting with dyspepsia, epigastric discomfort, or other nonspecific gastrointestinal symptoms potentially related to *H. pylori*. In this frontline capacity, GPs are responsible for initiating a “test-and-treat” approach - testing for *H. pylori *and starting appropriate therapy in those who test positive, especially in the absence of alarm symptoms [10]. Early GP intervention promotes ulcer healing, symptom resolution, and a reduction in complications such as bleeding or gastric cancer.

Additionally, GPs serve an educational function, counseling patients on hygiene practices to prevent intrafamilial transmission and stressing the importance of medication adherence to ensure successful eradication. Given these responsibilities, it is crucial that GPs possess up-to-date knowledge of *H. pylori *epidemiology, transmission, diagnosis, and treatment strategies. They should also be aware of emerging challenges such as antibiotic resistance. However, evidence from various countries suggests that knowledge and practice among physicians can be inconsistent. For instance, a study of GPs in Mexico found poor adherence to management guidelines, with frequent reliance on serological testing and inconsistent confirmation of eradication post-treatment [12]. This suggests that some practitioners may not be fully aware of the latest evidence-based recommendations.

In contrast, a recent study in Saudi Arabia found generally good awareness of *H. pylori *among physicians across multiple specialties, reporting an average knowledge score of 77% [13]. Internal medicine specialists tended to score higher than primary care physicians, and knowledge levels varied by region and years of experience [13].

Given the high global prevalence of* H. pylori *and its established role in gastric carcinogenesis, particularly in resource-limited settings, it is critical to understand how frontline providers manage the infection. This study aims to assess the knowledge, attitudes, and treatment practices of GPs in Riyadh, Saudi Arabia, regarding *H. pylori *infection. By identifying gaps in clinical awareness and deviations from guideline-based management, the study seeks to support targeted educational initiatives and inform policy development. On a national level, these insights could enhance diagnostic accuracy and treatment outcomes in Saudi Arabia, where *H. pylori *remains highly prevalent. Globally, the findings contribute to the broader understanding of primary care practices in *H. pylori *management, highlighting areas for harmonization and reinforcing the role of GPs in reducing complications such as peptic ulcer disease and gastric cancer.

## Materials and methods

Study design

This study utilized a cross-sectional, questionnaire-based design to evaluate the knowledge, attitudes, and treatment practices of GPs regarding *H. pylori* infection.

Study setting and population

The study was conducted in Riyadh, Saudi Arabia, and targeted licensed GPs currently working in primary healthcare centers (PHCs) across government, private, and other healthcare sectors.

Sampling strategy

A quota sampling method was employed to ensure diverse and proportional representation from the main types of PHC settings in Riyadh, namely government-run, private, and other institutions (such as military or university-affiliated centers).

To construct the sampling frame, a preliminary listing of PHCs in Riyadh was compiled using Ministry of Health directories and private sector listings. The city was then geographically stratified into five administrative regions: North, South, East, West, and Central. Within each region, healthcare centers were randomly selected in proportion to the estimated number of centers and staffing sizes. Quota targets were established to reflect the expected distribution of GPs across sectors, aiming for approximately 40-50% from private centers, 40% from government centers, and 10% from other institutions.

GPs were approached either in person through site visits or digitally via WhatsApp and email groups organized by PHC administrators and professional networks. Eligible participants who met the inclusion criteria were invited to complete the questionnaire. Sampling continued until the predetermined quotas for each category were met, resulting in a final sample of 91 respondents.

Sample size

A total of 91 GPs completed the questionnaire and were included in the final analysis.

Inclusion and exclusion criteria

Only licensed GPs working in PHCs within Riyadh who were willing to participate and provide informed consent were included. Physicians not practicing as GPs, as well as those working outside of Riyadh, were excluded from the study.

Questionnaire development

A structured, bilingual (Arabic and English) questionnaire was developed based on previously validated instruments and relevant literature. It consisted of four domains: demographics, knowledge of *H. pylori*, clinical attitudes, and treatment practices. The questionnaire was reviewed by three clinical experts to ensure content and face validity (Appendix A).

Data collection procedure

Data collection was carried out over a four-week period. GPs were invited to participate either in person at PHCs or through an online link to a Google Forms version (Google LLC, Mountain View, California, United States) of the questionnaire. Each participant was given an informed consent form and a brief explanation of the study's purpose and confidentiality protocols. Responses were checked for completeness before inclusion in the final dataset, and duplicate entries were avoided by restricting submissions to one per email address or device.

Data analysis

All data were analyzed using IBM SPSS Statistics for Windows, Version 26.0 (Released 2018; IBM Corp., Armonk, NY, USA). Descriptive statistics, including frequencies and percentages, were used to summarize categorical variables. Associations between GP characteristics and their knowledge, attitudes, and practices were examined using chi-square (χ²) tests, with a p-value of less than 0.05 considered statistically significant.

Ethical considerations

The study received ethical approval from the Institutional Review Board (IRB) at Almaarefa University under approval number IRB24-117. Participation was voluntary, and informed consent was obtained from all respondents. Confidentiality was maintained by anonymizing all responses, and no identifiable information was collected or reported.

## Results

Table 1 presents the distribution of demographic characteristics, knowledge levels, attitudes, and treatment practices among the 91 GPs surveyed in Riyadh. Of the participants, 66 (72.5%) were female and 25 (27.5%) were male. With regard to workplace setting, 45 GPs (49.5%) were employed in private clinics, 38 (41.8%) in government facilities, and eight (8.8%) in other settings. In terms of professional experience, 35 GPs (38.5%) had more than 10 years of practice, while 28 (30.8%) had five to 10 years of experience, and another 28 (30.8%) had less than five years. When asked about their knowledge of H. pylori, 57 participants (62.6%) considered their knowledge up to date, whereas 34 (37.4%) did not. In terms of clinical attitude, 70 GPs (76.9%) reported that they treat H. pylori cases, while 21 (23.1%) did not. Regarding the choice of first-line treatment regimen, 62 GPs (68.1%) prescribed triple therapy, 27 (29.7%) prescribed quadruple therapy, and two (2.2%) used other regimens. As for treatment duration, 54 GPs (59.3%) reported prescribing a 14-day course, 32 (35.2%) prescribed a 10-day course, and five (5.5%) reported using other durations. 

**Table 1:** Overview of GPs’ demographics, knowledge, attitudes, and treatment practices related to H. pylori Infection GP, general practitioner

Variable		Parameter	Frequency (n = 91)	Percentages (%)
Demographics	Gender	Male	25	27.5
		Female	66	72.5
	Clinical setting	Government	38	41.8
		Private	45	49.5
		Other	8	8.8
	Years of experience	Less than five years	28	30.8
		Five to 10 years	28	30.8
		More than 10 years	35	38.5
Knowledge	Do you consider your knowledge up to date?	Yes	57	62.6
		No	34	37.4
Attitude	Do you treat *Helicobacter pylori *cases?	Yes	70	76.9
		No	21	23.1
Practice	Which drug regimen do you personally prescribe as a first line?	Triple	62	68.1
		Quadruple	27	29.7
		Other	2	2.2
	What is the duration of treatment you give for *H. pylori*?	10 days	32	35.2
		14 days	54	59.3
		Other	5	5.5

Table 2 explores the relationship between years of clinical experience and GPs’ knowledge, attitudes, and treatment practices related to H. pylori. A statistically significant association was found between years of experience and self-reported up-to-date knowledge (p = 0.042). Specifically, 27 GPs (77.1%) with more than 10 years of experience considered their knowledge current, compared to 17 (60.7%) with less than five years and 13 (46.4%) with five to 10 years of experience. Although no significant association was observed between experience and the attitude toward treating H. pylori (p = 0.141), 28 GPs (80%) in the >10 years group reported treating H. pylori cases, along with 24 (85.7%) in the five to 10 years group and 18 (64.3%) in the less than five years group.

**Table 2:** Relationship between years of experience and GPs’ knowledge, attitudes, and practices toward H. pylori GP, general practitioner

Variable			Years of experience			p value (χ²)
			Less than five years (F, %)	Five to 10 years (F, %)	More than 10. years (F, %)	
Knowledge	Do you consider your knowledge up to date?	Yes	17 (60.7%)	13 (46.4%)	27 (77.1%)	0.042 (6.33)
		No	11 (39.3%)	15 (53.6%)	8 (22.9%)	
Attitude	Do you treat *Helicobacter pylori* cases?	Yes	18 (64.3%)	24 (85.7%)	28 (80%)	0.141 (3.92)
		No	10 (35.7%)	4 (14.3%)	7 (20%)	
Practice	Which drug regimen do you personally prescribe as a first line?	Triple	20 (71.4%)	10 (35.7%)	32 (91.4%)	0.001 (22.95)
		Quadruple	7 (25%)	17 (60.7%)	3 (8.6%)	
		Other	1 (3.6%)	1 (3.6%)	0	
	What is the duration of treatment you give for *H. pylori*?	10 days	7 (25%)	9 (32.1%)	16 (45%)	0.328 (4.62)
		14 days	18 (64.3%)	18 (64.3%)	18 (51.4%)	
		Other	3 (10.7%)	1 (3.6%)	1 (2.9%)	

A highly significant association emerged between years of experience and the choice of first-line drug regimen (p = 0.001). The most commonly prescribed regimen was triple therapy, typically combining a PPI, clarithromycin, and amoxicillin or metronidazole, used by 62 participants (68.1%). Quadruple therapy, usually a PPI, bismuth, tetracycline, and metronidazole, was used by 27 GPs (29.7%), while two respondents reported alternative regimens, including sequential therapy or levofloxacin-based combinations. Triple therapy was favored by 32 GPs (91.4%) with more than 10 years of experience, 20 (71.4%) in the less than five years group, and only 10 (35.7%) among those with five to 10 years. Quadruple therapy was most common among the five to 10 years group, with 17 GPs (60.7%) prescribing it.

Regarding treatment duration, although the association with experience was not statistically significant (p = 0.328), 16 GPs (45%) with over 10 years of experience prescribed a 10-day regimen, compared to nine (32.1%) in the five to 10 years group and seven (25%) in the less experienced group. Across all experience levels, a 14-day treatment course remained the most frequently prescribed duration, with 54 GPs (59.3%) using this regimen overall.

Table 3 examines the relationship between the clinical practice setting (government, private, or other) and GPs’ knowledge, attitudes, and treatment practices regarding H. pylori. In terms of knowledge, 25 GPs (65.8%) from government centers, 29 (64.4%) from private clinics, and three (37.5%) from other settings reported having up-to-date knowledge; however, this association was not statistically significant (p = 0.304). A significant association was observed in attitudes toward treating H. pylori (p = 0.006), with 41 GPs (91.1%) in private clinics reporting treatment of H. pylori cases, compared to 24 (63.2%) in government centers and five (62.5%) in other settings. Regarding treatment practices, a significant association existed between clinical setting and choice of first-line drug regimen (p = 0.007). Triple therapy was most commonly prescribed in government centers (33 GPs, 86.8%), whereas its use was lower in private clinics (25 GPs, 55.6%) and other settings (four GPs, 50%). Quadruple therapy was more frequently prescribed in private clinics (19 GPs, 42.2%) than in government (five GPs, 13.2%) or other centers (three GPs, 37.5%). Lastly, although not statistically significant (p = 0.086), a 14-day treatment duration was reported by 28 GPs (73.7%) in government settings, 21 (46.7%) in private clinics, and five (62.5%) in other centers. In contrast, a 10-day regimen was more commonly used in private clinics (22 GPs, 48.9%) than in government centers (eight GPs, 21.1%).

**Table 3:** Association between clinical setting and GPs’ knowledge, attitudes, and practices regarding H. pylori GP, general practitioner

Variable			Clinical setting			p value (χ²)
			Government (F, %)	Private (F, %)	Other (F, %)	
Knowledge	Do you consider your knowledge up to date?	Yes	25 (65.8%)	29 (64.4%)	3 (37.5%)	0.304 (2.38)
		No	13 (34.2%)	16 (35.6%)	5 (62.5%)	
Attitude	Do you treat *Helicobacter pylori *cases?	Yes	24 (63.2%)	41 (91.1%)	5 (7.1%)	0.006 (10.1)
		No	14 (36.8%)	4 (8.9%)	3 (37.5%)	
Practice	Which drug regimen do you personally prescribe as a first line?	Triple	33 (86.8%)	25 (55.6%)	4 (50%)	0.007 (14.12)
		Quadruple	5 (13.2%)	19 (42.2%)	3 (37.5%)	
		Other	0	1 (2.2%)	1 (12.5%)	
	What is the duration of treatment you give for *H. pylori*?	10 days	8 (21.1%)	22 (48.9%)	2 (25%)	0.086 (8.155)
		14 days	28 (73.7%)	21 (46.7%)	5 (62.5%)	
		Other	2 (5.3%)	2 (4.4%)	1 (12.5%)	

## Discussion

This survey of 91 GPs in Riyadh revealed notable gaps and variations in knowledge, attitudes, and practices related to *H. pylori *management. The sample was predominantly female (72.5%) and included GPs from both government and private primary care centers, with a balanced distribution of clinical experience. About two-thirds (62.6%) of participants reported having up-to-date knowledge of *H. pylori*, while the remaining 37.4% acknowledged gaps in their understanding. This self-reported deficit is concerning given that the majority (76.9%) actively manage *H. pylori *cases, underscoring the need to ensure clinicians are equipped with current knowledge and best practices.

Treatment patterns showed that 68.1% of GPs preferred triple therapy as a first-line regimen, while 29.7% opted for quadruple therapy. Regarding treatment duration, 59.3% prescribed a 14-day course consistent with global recommendations, yet a substantial 35.2% still favored a shorter 10-day regimen. These findings indicate variability in guideline application, which could impact treatment effectiveness and contribute to antibiotic resistance.

Significant differences emerged when comparing knowledge and practice across clinical experience levels. GPs with over 10 years of experience were more likely to report up-to-date knowledge (77.1%) than those with five to 10 years (46.4%) or less than five years (60.7%) (p = 0.042). Interestingly, the most experienced physicians were also more inclined to prescribe triple therapy (91.4%), whereas mid-career GPs (five to 10 years) favored quadruple regimens (60.7%), perhaps reflecting closer alignment with evolving international guidelines. Although not statistically significant, treatment duration trends also varied with experience: 45% of the most experienced physicians prescribed 10-day courses, while younger GPs more often recommended 14-day regimens.

Clinical setting influenced management practices significantly. GPs in private clinics were more likely to treat *H. pylori *cases themselves (91.1%) than those in government centers (63.2%) (p = 0.006), likely reflecting differences in specialist access, autonomy, or patient expectations. Additionally, private sector GPs more frequently prescribed quadruple therapy (42.2%) compared to their government counterparts (13.2%) (p = 0.007), suggesting greater adherence to evolving treatment recommendations or better medication access. Yet, 48.9% of private GPs prescribed 10-day regimens compared to only 21.1% in government settings, possibly influenced by cost, patient compliance, or routine practice. While differences in knowledge between sectors were not statistically significant, variations in treatment behaviors highlight the need for unified guidelines and equitable resource access.

These findings align with previous regional and international studies. Hakami et al. [13] documented variable *H. pylori* knowledge across physician specialties in Saudi Arabia, with internal medicine specialists scoring higher than primary care doctors, and noted the absence of unified local guidelines. Similarly, Cano-Contreras et al. [12] reported low guideline adherence among Mexican GPs, including reliance on outdated diagnostics and inconsistent follow-up. Our results echo these patterns, particularly the ongoing preference for triple therapy and variable treatment durations among Riyadh-based GPs.

Internationally, Malek et al. [8] identified limited public awareness and poor treatment-seeking behavior in the UAE, compounding challenges for physicians managing *H. pylori *infections. Al-Hussaini et al. [2] and Hooi et al. [1] documented high *H. pylori* prevalence in the Middle East, underscoring the vital role of frontline practitioners in early detection, appropriate treatment, and patient education.

From a public health standpoint, these findings carry significant implications. *H. pylori *is among the most prevalent infections worldwide, with established links to peptic ulcer disease and gastric cancer. Plummer et al. [5] and Sung et al. [6] emphasize its role as a major risk factor for non-cardia gastric cancer, which causes substantial global morbidity and mortality. In Saudi Arabia, where infection rates are high even among asymptomatic individuals [2], GPs play a crucial role in early diagnosis, effective treatment, and educating patients. Gaps in knowledge and inconsistent practices identified here could undermine eradication efforts, contribute to antibiotic resistance, and delay prevention of severe complications.

GPs also serve as primary educators for patients. Studies like Malek et al. [8] show that misinformation and reliance on traditional remedies remain widespread. Physicians who are confident and current in their knowledge are better positioned to guide patients on transmission, risks, and treatment adherence. The fact that nearly 40% of GPs in this study self-identified as not up-to-date highlights the urgent need for ongoing medical education to support this educational role.

Limitations

This study has limitations. The sample size was modest and limited to PHCs in Riyadh, which may not reflect practices in other Saudi regions, especially rural areas. Reliance on self-reported data introduces potential response bias, as actual prescribing behaviors were not verified through medical records. Additionally, knowledge assessment was based on subjective self-evaluation rather than objective testing. Future research should include larger, more geographically diverse samples, use case-based assessments to evaluate knowledge, and review medical records to compare reported versus actual practices. Qualitative studies could further explore factors influencing prescribing habits, such as patient preferences, drug availability, or institutional policies. Developing and evaluating targeted interventions - workshops, clinical algorithms, or national guidelines - will be essential for translating these findings into improved clinical outcomes.

Another limitation is the self-reported nature of data, which is susceptible to recall and social desirability biases. Participants may have overestimated guideline adherence or misremembered treatment regimens. To minimize this, the questionnaire was anonymous, and confidentiality was emphasized, encouraging honesty. Future studies might incorporate prescription audits or case vignettes to improve objectivity.

## Conclusions

This study highlights significant variations in the knowledge, attitudes, and treatment practices of GPs in Riyadh regarding *H. pylori* infection. Although most GPs showed willingness to update their knowledge and a general awareness of treatment protocols, substantial gaps remain in regimen selection, treatment duration, and self-reported knowledge. Clinical experience and practice setting significantly influenced these patterns. Given the high prevalence of *H. pylori* in Saudi Arabia and its serious health risks, especially gastric cancer, strengthening guideline dissemination, promoting continuous medical education, and standardizing management in primary care are essential. Enhancing GPs’ capacity to provide evidence-based care will be crucial for improving eradication rates, reducing complications, and advancing public health outcomes in the region.

## Appendices

Appendix A 

**Table 4:** Study questionnaire

Variable		Parameter
Demographics	Gender	Male
		Female
	Clinical setting	Government
		Private
		Other
	Years of experience	Less than five years
		Five to 10 years
		More than 10 years
Knowledge	Do you consider your knowledge up to date?	Yes
		No
Attitude	Do you treat *Helicobacter pylori* cases?	Yes
		No
Practice	Which drug regimen do you personally prescribe as a first line?	Triple
		Quadruple
		Other
	What is the duration of treatment you give for *Helicobacter pylori*?	10 days
		14 days
		Other
